# From a culture of caution to a culture of confidence: facilitating the good governance of administrative data in the UK

**DOI:** 10.23889/ijpds.v1i1.380

**Published:** 2017-04-19

**Authors:** Leslie Stevens, Graeme Laurie

**Affiliations:** 1 University of Edinburgh; 2 University of Edinburgh School of Law

## Objectives

This talk unpacks the culture of caution surrounding the use and sharing of administrative data in the UK and suggests the adoption of the authors' novel decision-making tool and organisational strategy based on the public interest, to achieve good governance. Administrative data, which implicate all public sector data, are in constant demand -to be shared for `joined-up' services, used as evidence in Government inquiries and for research purposes. These demands are often made on the basis that they serve `the public interest' but public authorities are without the decision-making tools to make proportionate decisions outwith narrow and risk-averse interpretations of legal requirements. Public authorities are operating within a `culture of caution', fuelled by misperceptions of what the law does or does not require for data to be used/shared `in the public interest'; uncertainties regarding incentives for data sharing; perceived controversies if something `goes wrong'; and imbalanced assessment of risks without robust assessment of potential public interests to be served or the potential `harm' from not sharing data.

## Approach

This discussion is substantiated by reference to major contributions to this field (e.g. Law Commission Report on data sharing in 2014; Thomas and Walport's data sharing review in 2008 etc.) and to the authors' engagement with the administrative data community as part of the legal work package to the Administrative Data Research Centre Scotland. 

## Results

The research reveals that public authorities exhibit extreme hesitance to undertake data sharing initiatives for reasons including:

- misperceptions of the law (due to legal complexity, lack of legal precedent and authoritative guidance on data sharing)
- lack of resources and expertise to manage increasing demands to use/share data
- individuals fear reprisal if something `goes wrong' with data handling
- senior-management fear public backlash for new uses of data and organisational reputational damage
- no understanding of the incentives to share data if there is no `direct' benefit to the public authority in question.

## Conclusion

We conclude by focussing on how to overcome the culture of caution, to one of confidence. We suggest the adoption of our decision-making matrix to help data custodians distinguish between real versus perceived barriers to data sharing (i.e. dispelling legal myths and identifying areas where changes can be made). We also introduce strategic solutions in our public interest mandate which entails overt commitment to use public sector data when it is in the public interest to do so.

